# Health care workers’ perspectives on care for patients with injection drug use associated infective endocarditis (IDU-IE)

**DOI:** 10.1186/s12913-022-08121-z

**Published:** 2022-05-31

**Authors:** Saira Butt, Mitchell McClean, Jane Turner, Sarah Roth, Angela L. Rollins

**Affiliations:** 1 Division of Infectious Diseases, Indiana University School of Medicine, 545 Barnhill Drive, Suite EH 421, Indianapolis, IN 46202 USA; 2 Regenstrief Institute, Indianapolis, USA; 3 Department of Psychology, Indiana University-Purdue University, Indianapolis, IN USA

**Keywords:** Infective endocarditis, Substance use disorder, Injection drug use, Endocarditis, Medication for opioid use disorder, Addiction medicine

## Abstract

**Background:**

Despite high morbidity and mortality, patients with injection drug use associated infective endocarditis (IDU-IE) lack standardized care, and experience prolonged hospitalization and variable substance use disorder (SUD) management.

Our study’s objective was to elicit perspectives of health care workers (HCWs) who deliver care to this population by understanding their perceived patient, provider, and system-level resources and barriers.

**Methods:**

This qualitative study included interviews of HCWs providing care to patients with IDU-IE from January 2017 to December 2019 at a single Midwest academic center. Based on electronic medical record queries to determine high and low rates of referral to SUD treatment, HCWs were selected using stratified random sampling followed by convenience sampling of non-physician HCWs and a patient. Study participants were recruited via email and verbal consent was obtained. The final sample included 11 hospitalists, 3 specialists (including 2 cardiovascular surgery providers), 3 case managers, 2 social workers, 1 nurse, and 1 patient. Qualitative semi-structured interviews explored challenges and resources related to caring for this population. Qualitative Data Analysis (QDA) Minor Lite was used for thematic data using an inductive approach.

**Results:**

Three major thematic categories emerged relative to patient-level barriers (e.g., pain control, difficult patient interactions, social determinants of health), provider-level barriers (e.g., inequity, expectations for recovery, varying levels of hope, communication style, prescribing medication for SUD), and system-level barriers (e.g., repeat surgery, placement, resources for SUD and mental health). The need to address underlying SUD was a prominent theme.

**Conclusion:**

Practical steps we can take to improve treatment for this population include training and coaching HCWs on a more person-centered approach to communication and transparent decision-making around pain management, surgery decisions, and expectations for SUD treatment.

**Supplementary Information:**

The online version contains supplementary material available at 10.1186/s12913-022-08121-z.

## Background

Injection drug use (IDU) increases the risk of infective endocarditis (IE), and is an independent risk factor for diminished survival from IE [1]. After a twelve-fold increase in IDU-related infective endocarditis (IDU-IE) admissions since 2013, IDU-IE now accounts for 10% of annual hospitalizations for IE in the United States [2]. Almost half of patients with IDU-IE undergo valve surgery [3]. Although recent studies have shown oral antibiotics are non-inferior to intravenous (IV) antibiotics [4, 5], most patients with IDU-IE are currently managed with weeks of intravenous (IV) antibiotics at a hospital or in a specialized facility based on the guidelines [6]. The most common cause of death in IDU-IE is usually a recurrent episode of IE [3]. A recent survey of cardiac surgeons revealed willingness to operate on only 26% of patients with recurrent IDU-IE versus 93% of patients with recurrent non-IDU-IE [7], indicating a potential inequity of treatment that warrants further exploration.

In a national survey, the majority of the infectious diseases physicians highlighted the complexity of management of IDU-related infections due to structural barriers to conventional management including a lack of dedicated multidisciplinary addiction services [8]. Long-term treatments, including medications for opioid use disorder (MOUD), are critical for the recovery of patients with substance use disorder (SUD) [9]. Despite lengthy hospital stays and surgery, utilization of MOUD and linkage to SUD care for patients with IDU-IE is inadequate [2, 10, 11]. Both health care systems issues and HCWs attitudes’ have at times contributed to the challenges faced by people with IDU-IE. When surveyed, patients with a history of IDU perceived multiple system issues including poor pain management, premature discharge, and lack of appropriate outpatient follow-up appointments [12]. Studies of HCWs’ perspectives’ demonstrated misinformation and stigma towards patients with IDU and the provision of MOUD poses barriers to treatment access. HCWs have cited the lack of patient motivation in recovery as a source of dissatisfaction when treating this population [13]. When patients with IDU-IE who have undergone valve surgery develop recurrent infections, HCWs attitudes’ and approaches towards repeat valve surgeries varied widely in one study [14]. The same group explored the experiences of HCWs managing IDU-IE in both inpatient and outpatient settings and concluded shortcomings in care at critical transition points may explain the return to drug use, rehospitalizations, and death [15]. Our study aims to expand this literature by documenting the perspectives of HCWs regarding a broad range of challenges and resources while treating IDU-IE during a critical hospitalization. Ultimately, these perspectives could potentially inform improvements in how we provide care to this patient population with complex needs.

## Methods

### Participant sampling and recruitment

This study took place at a large academic health care system in the Midwest providing inpatient care for IDU-IE. First, we sampled inpatient HCWs based on services provided to patients with IDU-IE. To select HCWs, we reviewed electronic medical records data of all patients admitted from January 2017 to December 2019 with an ICD-10 diagnosis of IE (I33, I33.9, I138, I39, B37.6). Then, to approximate IDU in the absence of a specific ICD-10 code, we selected patients with IE who also had at least one ICD-10 diagnosis of hepatitis C (B17.1, B18.2, Z22.52) or drug use (Z72.2, Z86.41) or mental or behavioral disorder (F11, F14, F15, F19) codes [16]. From this list of patients, we also obtained the names of the HCWs who requested a SUD medicine consult to assess which HCWs were most frequently taking action to address SUD.

We used a stratified random sampling approach by dividing the resulting provider population into lists by HCW type (physicians and advance practice providers [APP] hospitalists, and APP specialists [cardiovascular surgery and SUD medicine], and frequency of referral for SUD treatment (2 or more versus 1 or none). We randomly recruited HCWs until the goals for each stratum were achieved, with the highest priority on exhausting lists of high-frequency groups and physician hospitalists first. Based on these HCWs’ recommendations, we used convenience sampling to interview patients and other HCWs (case managers, social workers, and nurses) involved in the care of this population at the hospital. Local institutional review board approval was obtained.

### Interview and data collection

Based on our literature review, we developed a semi-structured interview guide to elicit the diversity of attitudes and approaches toward treating patients with IDU-IE. We revised the guide based on the initial themes and feedback. (See online Additional file 1) Study participants were recruited via email by the principal investigator (PI). Based on expressed interest in participation, verbal consent was obtained for the qualitative interview. The participation was voluntary and uncompensated. Interviews lasting approximately 30 minutes were conducted and recorded remotely using the health system’s secure videoconferencing platform Zoom (San Jose, CA). Interview video files were stored in the secure Microsoft Teams application (Redmond, WA). Interviews were transcribed, de-identified, and checked for accuracy.

### Qualitative data analysis

Transcribed interviews were imported into Qualitative Data Analysis (QDA) Minor Lite software for data analysis management and analyzed using thematic analysis techniques using an inductive approach [17]. The analytics team included an infectious disease physician (SB), a health services researcher (AR), two infectious diseases fellows (MM and JT), and a research assistant experienced in qualitative analysis (SR). Two members of the analytics team, SB, and AR conducted a close reading and review of all transcripts noting what stood out as meaningful about the research question, and together developed an initial codebook. The codebook was tested and refined by all members of the analytic team iteratively reading, coding defining, and coming to a coding consensus for four interviews. Once a final codebook was established, the team divided up and coded the remaining interviews. Ten percent of the interviews were coded in pairs. The team met to discuss and resolve discrepancies and arrived at a final consensus for codes. Members of the team next organized codes into larger themes, meeting regularly to review, discuss and refine the names and definitions of each theme and ensure that the final themes accurately represented the data set as a whole.

## Results

The final sample included 9 physician hospitalists, 2 APP hospitalists, 3 APP specialists (including cardiovascular surgery providers), 1 nurse, 3 case managers, 2 social workers, and one patient. Respondents were predominantly male (*N*=12, 57%), White (*N*=15, 71%), non-Hispanic (*N*=20, 95%), and had a mean age of 42 (SD= 9) years. Respondents described a wide range of challenges and few resources for successfully treating IDU-IE. These challenges and resources were described relative to patient-level barriers, provider-level barriers, and system-level barriers. Prominent ideas within each of these broad thematic categories are described below with illustrating quotes.

### Patient-level barriers: attitudes, actions, and social contexts

Respondents described several challenges related to the patients themselves and their social contexts, primarily centered around the difficulties presented because of SUD.

For instance, common among the hospitalist respondents was the idea that pain control in a surgical patient with SUD was difficult, primarily attributed as a patient-level barrier, as opposed to being indicated as a limitation of treatments available.

*Setting appropriate expectations for pain management is exceedingly important and challenging because, especially those who end up getting valve surgery, they're going to have pain. And managing that pain is very challenging because it's real, but yet they're also addicts. And then supporting them through this forced sobriety and the stresses that causes. It's much easier when the patient is frank and honest about their addiction and always much more challenging when they try to hide or pretend that they are not an addict.*

Some hospitalists discussed how disrespectful attitudes of patients make it difficult to treat this population, making patient interactions difficult.

*Getting nurses and other physicians to treat patients respectfully is difficult because it's so easy to fall into the trap of giving back the disrespect they give to you.*

They are so manipulative in the midst of their addiction, it’s very painful to hope and care for these people who then let you down.

Many respondents spoke about detrimental patient-level social factors that impede the course of recovery, such as lack of transportation to access care, poverty, homelessness, lack of employment, and lack of health insurance. The challenge of the whole family suffering from SUD was discussed as well. The social worker discussed challenges around safe living arrangements after discharge to the community.

*The housing situation is so tumultuous and to get on any sort of program is a long waiting list, so they’re just stuck, and providers think, well why don’t they just move? And why don’t they just change their friends? Where are they going to move to? They’re going to go to a homeless shelter, where there are drugs. Or they’re going to be on the street where they have to maybe do things in order to earn money so you’re just adding more trauma.*

One of the hospitalists described the indelible social situation as

Some people are just really in a bad situation and have been since birth.

Some social circumstances were described as beneficial to recovery, such as motivation for sobriety because of their children or having family members or HCWs who were supportive of recovery, as described by one of the hospitalists.

*The patients that had good outcomes were ones that ended up making friends with their nurses and addiction therapists and, once you gain that rapport, that makes a critical difference. Because there are some people with a family member, that's a good support, that's willing to help them get a second chance in recovery, that makes a difference.*

### Provider-level barriers: expectations, understanding, and comfort with treating a person with SUD

Respondents observed that patients with SUD are treated differently compared to other patient populations. One of the hospitalists described an early automatic bias before the patient encounter.

*When you have a patient with drug addiction, there’s an automatic bias that gets planted as a seed in your head before you even see the patient, just hearing about their case from the emergency room doctor.*

One of the social workers referred to the notion that providers expected patients with IDU-IE to quit promptly when they are treated for such a serious condition.

*People have such a high expectation for them to change their lives first go around and not ever screw up, not ever relapse, and I think that is just not possible.*

One of the hospitalists described how the society including providers views this population.

*If we don’t judge the smoker who fails to quit smoking, why are we so judgmental of the meth addict and the heroin abuser? If your aunt can’t quit smoking after quitting seven times, you're not going to not invite her to Thanksgiving? We have decided as a society that this is unacceptable behavior. And if you can’t quit, there’s something wrong with you. And it's not my problem to fix it, it’s your problem. And unfortunately, many physicians have not had a good enough exposure to addiction training, and they haven’t had a good enough exposure to the successes of addiction counseling. So, they have this mindset that everyone’s a lost cause.*

A cardiovascular provider explained how the expectation is discussed with the patient.

*I probably have a scripted speech that you know why you’re here, and if you continue to do that, you’re going to be back here no matter what we do. And so, the only option that you have is abstinence …and I know that’s difficult because depending on who you use with or use around, you may be talking about trying to sever ties with family members, friends, or loved ones, and that’s never easy but that’s the only way this is going to get better.*

One hospitalist explained how HCWs’ expectations could negatively influence patients’ attitudes to recovery.

*A lot of patients having doubts or having resistance towards seeking treatment is that they've had it worded to them in the wrong way, in the past, by other people where they say, this is something you need to get over and you’re just not trying hard enough.*

Respondents felt frustrated or hopeless after repetitive negative outcomes of IDU-IE and one of the hospitalists described the lack of exposure to successful cases.

I don’t know of someone who has recovered. The ones that we tend to see are the ones that don’t do well.

Although much less common, HCWs who seemed most hopeful about treatment were those who had previous personal experience with SUD. They seemed comfortable with the idea that patients may have recurrent periods of relapse. One of the hospitalists described his personal experience leading to hopefulness.

*One of the things that helped me is I had a life experience where my family was touched with addiction … through that personal experience, you recognize there is recovery and there is a better life on the other side. For many of us, since we only see the bad seeds, who fall back into their second or third round, who tell us they're going to quit and then come back with another round of endocarditis. It's easy to believe that they will never recover and there's no chance for a better life. Helping to remind ourselves, that no, people do recover and in fact, there is better life on the other side of this*.

There were different ways HCWs approached difficult conversations with patients. Many HCWs described more of a need for setting hard expectations and boundaries but others described the value of communicating in a more personal way. Many HCWs described communication with the patients almost with a sense of dread and frustration. To combat numerous problems they perceived in serving the population, they endorsed various forms of a more structured set of expectations, boundaries, or rules for communication with patients. For example, one of the hospitalists reported a more hardline style of limiting patient choices, “*My approach is to confront them with the facts*.” Likewise, a nurse spoke of having written expectations (a pamphlet) and a social worker mentioned the importance of care contracts dictating behavioral boundaries with some patients. Others, however, approached difficult conversations very differently.

One case manager described a gentle communication approach.

I’m still very gentle about it because not being gentle can make them defensive, inadvertently. But they know they’ve got a problem.

One of the hospitalists described the significance of sitting down and listening to the patients.

*I listen to them. I make sure I sit down in their room and sit down in the chair, the windowsill beside them. A lot of people feel they’re not heard, and just discarded, because they’re addicts. I’ve gotten several comments like thanks for listening and talking with me about this.*

Another social worker was also invested in listening to the patient’s narrative.

*And a lot of times when I sit down and talk to these patients to figure out, when did it start for you and why did it start for you? At what age? And what did you start with? … piecing together kind of that puzzle*.

A patient respondent described two approaches to difficult conversations.

*I had one nurse…she wasn’t having it, she was very firm with me, she put her foot down, it was shocking to me at first. Then she came back and she softened it and she personalized my experience; I had some pictures up in my hospital room of my kids who had been removed from my care and she turned and looked at those pictures and she said, “who are these children?” And she connected with me after she set her clear boundaries and let me know that I couldn’t talk that way to her and I wasn’t going to act that way or demand pain meds ... that was a life-changing moment for me because you’re getting endocarditis patients at their worst. I didn’t feel like a human, I didn’t feel like a person, I was angry, I didn’t care if I lived or not. And her, having the conversation with me, regarding my children, helped save my life*.

HCWs reported a lack of experience and confidence in prescribing MOUD. Most continued MOUD if the patient was already on it but did not initiate MOUD in the hospital especially if no access was available outside the hospital on discharge. Multiple HCWs expressed feelings of powerlessness when linking patients with outpatient SUD services.

*There are still a lot of gaps … they’re just dumped out there and I don’t think we have a good follow-up as far as ownership of these people. We’ve got a good multi-disciplinary team when they’re in the hospital but I think once they’re out there, there’s no one that really has ownership.*

We can tell them, you just need to go to a methadone clinic but how that actually happens is like 20 more steps.

### System-level challenges and resources

The respondents described system-level challenges when treating IDU-IE, including the accessibility of appropriate housing placements or mental health services at discharge. Other concerns centered around systems issues with IE treatment itself. For example, many respondents discussed how this population is treated in a different way when surgery practices are considering repeat surgeries. The example was described by one of the hospitalists.

*If a 60-year-old guy reinfects his valve with strep mitis, they’re taking him to the OR (operating room) the next day and if an IV drug user gets staph aureus 6 months later, they’re not going to rush into that. So, that is a huge, big issue that I really don’t have a whole lot of control on but yeah, they definitely get treated differently*.

Varied approaches to repeat surgeries adapted by the surgeons were explained by one of the cardiovascular surgery providers.

*Our surgeons have different perspectives and some of our surgeons are more strict or less strict about saying no you do or don’t get a re-do operation. You don’t get a re-do operation if you tell me that you’ve re-used. We have some surgeons that are seemingly more forgiving and say well you’re back, let’s fix it again.*

HCWs acknowledged and praised the resources provided by social workers, the SUD medicine team, and case managers but there were still challenges to completing the follow-up treatment plans successfully. For example, one of the social workers noted that patients had to make a personal effort for linkage into care, and housing resources were not readily available.

*Like doctors, they legitimately asked me, so you think you can find them a place to go before they discharge. And I just want to laugh, let me check I think I have an extra apartment here, in my pocket. I would love to be able to set them up with housing! But, unfortunately, just the bigger picture, it’s just not possible.*

*I felt the medical team’s attitude shifted when we were more consistently able to* *find a placement*.

Respondents appreciated the SUD medicine team’s ability to manage patients’ pain medications and MOUD. They were able to link the patients to recovery for the short term but were challenged by a lack of long-term engagement and acknowledged that relapse was common.

*Sometimes we get help from chemical dependency, they write for suboxone and methadone, but the real challenge comes in when we have to discharge these patients to rehab. Most of these places don't have doctors who can write for this medication. So, we end up sending them out either on oxycodone or norco, those kinds of medications. That's definitely a challenge because not all providers can write for methadone or suboxone*.

Respondents described the challenge of insufficient mental health resources outside the hospital.

*We don’t have enough mental health and addiction resources, outside of the hospital. I try to be more optimistic at the beginning, but they’re fighting an uphill battle for sure*.

## Discussion

Study responses described many complex challenges of treating patients with IDU-IE. Themes emerged with respect to the patient, provider, and system factors, with some themes varying by respondent type. However, the most prominent issue weaved throughout patient, provider, and system factors and across respondents was the challenge of addressing the underlying SUD.

Within patient-level factors, HCWs expressed frustration with managing pain and having challenging interactions with patients admitted for IDU-IE, as well as managing difficulties that many patients face with regard to negative social determinants of health that serve as barriers to both SUD and IE recovery. Indeed, prior literature on pain management in populations with SUD indicated that patients also struggled with poverty and mental health. In one study, dealing with a patient’s social rather than medical needs seemed to go beyond provider frustration and was found to contribute to burnout [18].

The theme of communication between patients and HCWs was also prominent across patient and provider-level factors. Perhaps the most striking theme in this area was contrasting approaches to communication (i.e., curious, active listening versus firm). Some HCWs reported relying on confrontation and boundary setting when discussing treatment plans with the patient. However, others described a gentler approach when setting expectations. Interestingly, the patient described the effectiveness of communication she experienced that balanced both healthy boundaries and warmth. A more person-centered and engaging approach is consistent with transtheoretical models of change embraced in patient care models where resistance to behavioral changes is common [19]. This strategy could be the first step to reaching this population at a personal level during a critical episode of care and help them participate and engage in their care.

HCWs expressed varying levels of frustration with patient relapse to SUD after discharge, with some recognizing the difficulty accessing needed MOUD and other supports that facilitate sobriety. Despite substantial challenges in social determinants of health, some respondents hoped that patients with IDU-IE would quit injecting drugs promptly after discharge from this critical, life-saving hospitalization. In a previous study regarding SUD, perceptions of patients’ high controllability over IDU contributed to the negative attitudes of HCWs [20]. Other HCWs in our study highlighted difficulties in communication with these patients and the power of being able to connect with patients on a personal level to prompt their pursuit of recovery at such a critical point in their illness.

Although less common, a few HCWs expressed outright hopefulness about the potential for SUD recovery and provided an interesting perspective using personal experience with SUD and recovery. For instance, knowing someone who had been in long-term recovery seemed to allow these respondents to have a more hopeful outlook on the treatment of SUD. They seemed more comfortable with the idea that patients may have recurrent periods of abstinence and use during the course of early recovery.

Similarly, in another study, HCWs who were more frequently in contact with people with IDU expressed more positive explicit attitudes towards these patients [21, 22]. Another study found that anesthesiologists with a personal history of SUD reported more positive attitudes toward patients suffering from these problems [23].

In general, our findings and the accompanying literature highlight the mostly unrealized potential of strategically exposing more HCWs to stories of SUD recovery in hopes that they can develop hopeful attitudes and communication techniques with patients that can facilitate SUD recovery.

On a system level, related to HCWs’ frustration and feelings of discomfort in dealing with patients with SUD, several HCWs highlighted the need for additional training on SUD. Previous literature shows that even short SUD medicine training programs can be effective in improving knowledge, skills, and attitudes [24]. Linkage to outpatient care for MOUD, mental health, and other social service needs (e.g., housing, transportation, employment) was also important according to many respondents, which is consistent with previous studies. Geographic proximity to services and lack of transportation to reach limited mental health and MOUD services are commonly cited in the literature as a barrier to treatment access [25–27].

Our study has some limitations, including experience from a single center hospital center and only one patient’s perspective. However, our findings are largely consistent with previous studies. Although most HCWs endorsed feelings of frustration regarding continued substance use, there were a few provider respondents whose prior experience with SUD recovery indicates that there might be ways to address pessimism toward this population. We identified several opportunities to provide more education to inpatient HCWs on SUD and its treatment and to improve comprehensive care for people with IDU-IE.

### Implications for practice improvements

These themes certainly inform how we might conceptualize improvements in caring for patients with IDU-IE. Practical steps might include improving provider communication skills to adopt active listening and patient engagement. These skills might enable transparent decision-making around pain management, surgery decisions, and expectations for MOUD while hospitalized. The themes from respondents certainly varied in terms of how structured or “loose” they felt that guidelines should be in how to make decisions for repeated surgeries, and this finding matched prior work by Hayden and Moore [13]. Other practical steps might include broader hospitalist education around SUD recovery and treatments, including hearing from patients who are in active recovery. Other broader system-level changes suggested by our respondents, such as the need to expand access to effective outpatient treatment for SUD and addressing social determinants of health, may take more concerted efforts on the part of healthcare system leaders and healthcare funders. For instance, an expansion of chronic care models, using a proactive population health approach and comprehensive care coordination to reduce social deterrents to care and engage patients in needed follow-up care [28–30] may be increasingly important for patients with complex chronic conditions like IDU-IE. However difficult, these changes could impact the outcomes and long-term costs of a complex and highly vulnerable patient population receiving expensive inpatient care.

## Conclusions

In this study, a range of HCW perspectives was identified regarding the challenges of providing care to patients with IDU-IE. Challenges included patient-level, provider-level, and systematic factors, with frequent and consistent concerns regarding the need to address the underlying SUD to adequately care for this population. Our paper highlights important factors that might be the focus of care improvement efforts, such as interventions to facilitate patient-provider communication and decision-making, particularly with regard to pain control, repeat surgeries, and expectations for SUD treatment.

## Supplementary Information

Additional file 1. (PDF 85 kb)

## Data Availability

The datasets used and/or analyzed during the current study are available from the corresponding author on reasonable request.

## Abbreviations

APP: Advance practice providers
CM: Case manager
HCWs: Healthcare workers
IDU: Injection drug use
IE: Infective endocarditis
IDU-IE: Injection drug use associated infective endocarditis
SUD: Substance use disorder
MOUD: Medications for opioid use disorder
PI: Principal investigator
QDA: Qualitative Data Analysis
SW: Social worker
