# Supplementary material for: Health care workers’ perspectives on care for patients with injection drug use associated infective endocarditis (IDU-IE)
Source: BMC Health Serv Res. 2022 May 31;22:719. doi: 10.1186/s12913-022-08121-z (PMC9153089; doi:10.1186/s12913-022-08121-z)
Supplement: Supplementary file 1 — Additional file 1. (PDF 85 kb) [file 12913_2022_8121_MOESM1_ESM.pdf]

Research Questions

1. Tell me about a recent patient that you cared for who was admitted with injection drug use associated infective endocarditis (IDU-IE). We are particularly interested in what you found to be challenging for this particular patient and how you made the recommendations for the type of care they needed?
2. Let's talk about what you typically do when you encounter a patient with IDU-IE. We are interested in the full range of your role: anything from assessment to treatment on the unit to coordinating discharge planning.
3. People have varying opinions regarding substance use disorder and treatment. What do you think is your primary role in treatment of patient with IE who has substance use disorder?
4. We talked a lot about your own thoughts and actions about treating this population. Let's switch gears and talk about how other health care workers treat this population. For instance, how do you think other health care workers conceptualize treatment for IDU-IE?
5. What suggestions do you have for improving our care for this population? We are interested in your take on what the ideal care model would involve?
